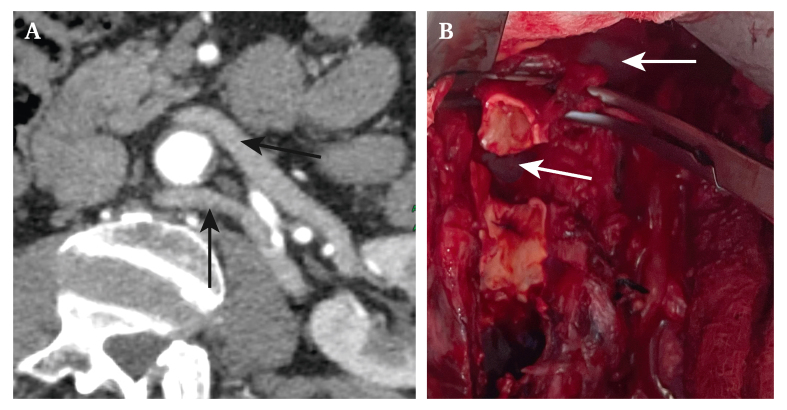# Circumaortic Left Renal Vein: A Challenge in Open Aortic Surgery

**DOI:** 10.1016/j.ejvsvf.2023.06.002

**Published:** 2023-07-06

**Authors:** Eduardo Arrea Salto, Abel Vélez Lomana

**Affiliations:** Vascular Surgery, Hospital Clinico Universitario Lozano Blesa, Zaragoza, Spain

A 62 year old man with a medical history of dyslipidaemia and tobacco use presented with lifestyle limiting right lower limb claudication due to a right common iliac artery occlusion. The imaging studies revealed a concomitant 40 mm aortic aneurysm and a circumaortic left renal vein (CLRV) (A, arrows). The patient underwent open surgery with an aortobifemoral bypass, using a 14 × 8 mm silver impregnated polyethylene terephthalate graft. CLRV poses a challenge in open aortic surgery, as the posterior limb travels obliquely behind the aorta and inserts caudally into the inferior vena cava. The aorta was transected at the aneurysm neck to prevent injury to the posterior left renal vein during the proximal anastomosis (B, arrows). The patient was discharged successfully after 10 days and remains complication free at one year follow up.

**Figure:**